# Twist-Controlled Second-Order Spatial Differentiation in a Three-Sheet Black Arsenic Stack

**DOI:** 10.3390/nano16201315

**Published:** 2026-10-18

**Authors:** Wenhao Xu, Shiyang Zhang, Lan Xu

**Affiliations:** 1 School of Physics and Chemistry, Hunan First Normal University, Changsha 410205, China; xulan@hnfnu.edu.cn; 2 Key Laboratory of Low-Dimensional Quantum Structures and Quantum Control of the Ministry of Education, Hunan Normal University, Changsha 410081, China

**Keywords:** optical spatial differentiation, photonic spin Hall effect, analog optical computing, black arsenic, twisted two-dimensional materials

## Abstract

We investigate transmission-mode second-order spatial differentiation in a stack of three twisted black-arsenic (b-As) conducting sheets separated by dielectric spacers. The symmetric twist provides a structural degree of freedom for controlling the normal-incidence Jones response and suppressing the zeroth-order helicity-converted background. Near the matching condition, the opposite-helicity transmission is dominated by a quadratic momentum-space response proportional to ${\left(k_{x}\pm ik_{y}\right)}^{2}$, giving conjugate phase windings with topological charges *ℓ* = ±2. The corresponding complex operator combines Cartesian and mixed second-order derivatives and produces second-order beam shaping and two-dimensional edge enhancement. Full transfer-matrix calculations confirm the expected real-space operation for both Gaussian and image inputs within the demonstrated numerical aperture. These results show how crystallographic twist and multilayer interference can be used to engineer polarization-selective spatial operators in anisotropic two-dimensional multilayers without subwavelength patterning.

## 1. Introduction

Optical analog computing has attracted sustained interest because mathematical operations can be performed directly on the spatial spectrum of an optical field, offering intrinsic parallelism and avoiding repeated optical–electronic conversion [1,2]. Spatial differentiation is one of the most useful operations in this context because local variations, boundaries, and fine structural details of an object are naturally encoded in its spatial derivatives. A broad range of optical platforms has therefore been developed for gradient extraction and edge enhancement, including flat-optical differentiators, multifunctional metasurfaces, dielectric edge-detection elements, quantum edge-imaging schemes, and Laplacian-type metasurfaces [3,4,5,6,7,8,9]. These developments have established that a properly engineered momentum-space transfer function can replace part of the numerical processing normally performed after image acquisition. Beyond first-order gradient operations, second-order differentiation is especially attractive for two-dimensional contour extraction because it can combine amplitude suppression around the spectral origin with a structured phase response, thereby connecting optical image processing with vortex and topological features of the transmitted or reflected field.

A complementary route to spatial differentiation is provided by the spin–orbit interaction of light and the photonic spin Hall effect (PSHE). The PSHE originates from polarization-dependent geometric phases and spin-dependent transverse momentum shifts, and has been investigated from both fundamental and application-oriented perspectives [10,11,12,13,14,15]. Because the spin-dependent response is naturally coupled to the transverse wave vector, polarization preselection and postselection can convert beam-shift physics into a spatial transfer function. This connection has been used to realize generalized spatial differentiation, Brewster-effect-based two-dimensional differentiation, and photonic spin-Hall differential microscopy [16,17,18]. Of particular relevance to the present work, second-order complex differentiation has been demonstrated in an anisotropic epsilon-near-zero slab, while normal-incidence topological differentiation has revealed the close relation between helicity conversion and phase winding [19,20]. Related studies have further extended PSHE-assisted spatial operations to Weyl and Dirac photonic systems [21,22]. Together with recent developments in spin-Hall microscopy and broader studies of the field [23,24,25], these works indicate that polarization conversion is not only a beam-shift phenomenon but also a useful mechanism for constructing spatial operators. Two-dimensional conducting sheets have also provided flexible platforms for tailoring the PSHE, as demonstrated in antimonene-assisted plasmonic structures and generic conducting-sheet interfaces [26,27].

Strongly anisotropic two-dimensional materials provide an additional degree of freedom because their in-plane optical response can be manipulated through crystallographic orientation. Rotation-dependent PSHE has, for example, been demonstrated in an anisotropic ReS_2_–graphene heterostructure, where the in-plane response can be tuned through the crystal orientation [28]. Effective surface-conductivity models have shown that anisotropic two-dimensional semiconductors can support highly direction-dependent optical responses [29]. Black arsenic (b-As), in particular, is a layered elemental semiconductor with pronounced in-plane anisotropy, as established by structural, optical, electrical, and dielectric studies [30,31,32]. This anisotropy makes b-As attractive for polarization-sensitive light–matter interactions. Our previous work considered the PSHE at a single b-As interface and showed that the anisotropic conductivity strongly affects the co- and cross-polarized reflection channels [33]. A different opportunity arises when several anisotropic sheets are spatially separated and relatively rotated. In twisted few-layer anisotropic two-dimensional crystals, the relative orientation of the layers and the Fabry–Pérot phase accumulated between them can jointly reshape the polarization response and the associated spin Hall shifts [34]. These observations suggest that twist should be viewed not merely as a way to vary the magnitude of anisotropy, but as a structural variable capable of reorganizing the total polarization-transfer response of a multilayer stack.

These developments motivate a more specific question: whether relative crystallographic twist can be used as a Jones-matching degree of freedom in an anisotropic multilayer, rather than only to modify the response of an individual interface. The generic helicity-2 quadratic response is already allowed in rotationally symmetric stratified systems through off-axis p/s splitting and the associated geometric phase [19], so the present contribution is not the introduction of a new $q^{2}e^{\pm 2i\varphi }$ mechanism. Instead, we use a symmetric three-sheet b-As stack to suppress the momentum-independent helicity-converted background through twist-controlled tensor compensation while the two spacer phases participate in the full multilayer matching. The three-sheet geometry is a convenient symmetric implementation rather than a unique or mathematically minimal route to tensor compensation. In this sense, the work extends established spin–orbit differentiation and twisted-anisotropic-sheet concepts by using crystallographic twist specifically as a control parameter for postselected Jones matching [20,33,34].

We derive the helicity-resolved transfer function of the structure and characterize its second-order momentum dependence, phase winding, numerical-aperture range, and real-space differentiation. A bare-stack control calculation is used to distinguish the generic dielectric contribution from the twist-controlled role of the b-As sheets. We also examine fabrication tolerance, conversion efficiency, and polarization postselection to delimit the practical operating regime. The purpose is therefore to establish twist-controlled Jones matching as a design principle for polarization-selective second-order processing, while quantifying its operating window and practical limitations.

## 2. Theoretical Model

**Figure 1:**
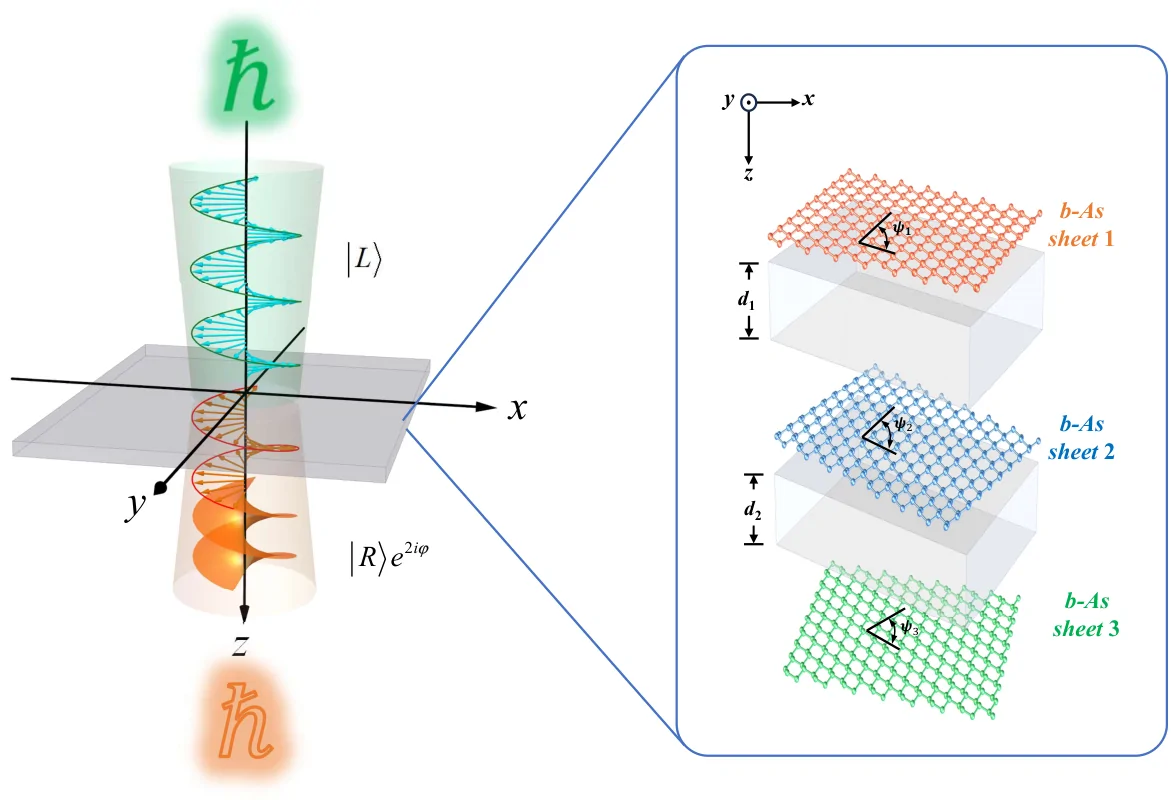
Schematic of the transmission-mode second-order spatial differentiator based on three spatially separated black-arsenic (b-As) sheets. A circularly polarized input field is normally incident on the twisted b-As stack, and the opposite-helicity transmitted component is selected as the output channel. The enlarged view shows the three anisotropic b-As sheets separated by dielectric spacers of thicknesses $d_{1}$ and $d_{2}$, with their in-plane principal axes oriented at $\psi_{1}$, $\psi_{2}$, and $\psi_{3}$, respectively. Black arrows indicate the laboratory coordinate axes and propagation direction; red, blue, and green identify b-As sheets 1–3, respectively, while the colored beam profiles schematically distinguish the incident and opposite-helicity output fields.

As illustrated in Figure 1, we consider three spatially separated black-arsenic (b-As) conducting sheets separated by two dielectric spacers. The incident and output half-spaces are air, $n_{0}=n_{3}=1$, while the two dielectric spacer regions have $n_{1}=n_{2}=n_{s}=3.4$. Each b-As layer is modeled as an optically thin conducting sheet described by a surface-conductivity tensor, so no independent geometrical sheet thickness enters the transfer matrix. The sheets are parallel to the laboratory xy plane, the central beam propagates along +z, and the in-plane principal axes are oriented at $\psi_{1}$, $\psi_{2}$, and $\psi_{3}$. The spacer thicknesses are denoted by $d_{1}$ and $d_{2}$. Throughout this work, the temporal dependence is exp(−iωt).

### 2.1. Anisotropic Surface Conductivity and Twist-Controlled Tensor Compensation

The in-plane optical response of each b-As sheet is described by the minimal effective anisotropic surface-conductivity model introduced for two-dimensional semiconducting materials [29]. The Drude term provides the intraband response, while the interband contribution is phenomenological; the provenance of the material-specific and model parameters used here is clarified in Section 3. In the principal-axis basis of an individual sheet, the conductivity tensor is

(1) $$\sigma_{0}=\left(\begin{array}{cc} \sigma_{x} & 0\\ 0 & \sigma_{y} \end{array}\right).$$

The two principal conductivities contain intraband and interband contributions,

(2) $$\sigma_{j}=\frac{ie^{2}n_{2D}}{m_{j}\left(\omega +i\Gamma \right)}+\sigma_{u}s_{j}\left[\Theta \left(\omega -\omega_{j}\right)+\frac{i}{\pi }\ln\left|\frac{\omega -\omega_{j}}{\omega +\omega_{j}}\right|\right],\;j=x,y,$$

where $n_{2D}$ is the sheet carrier density, $m_{j}$ is the direction-dependent effective carrier mass, Γ is the carrier-scattering rate, $\omega_{j}$ and $s_{j}$ describe the onset and strength of the interband contribution, respectively, and $\sigma_{u}=e^{2}/\left(4\hbar \right)$. The material parameters used below are specified in Section 3.

For sheet *i*, $\psi_{i}$ denotes the fixed in-plane orientation of the crystalline *x* principal axis, measured from the laboratory *x* axis toward *y*. Its conductivity tensor in the laboratory transverse basis is

(3) $$\sigma_{\psi_{i}}=U\left(\psi_{i}\right)\sigma_{0}U^{T}\left(\psi_{i}\right),\;U\left(\psi_{i}\right)=\left(\begin{array}{cc} \cos\psi_{i} & -\sin\psi_{i}\\ \sin\psi_{i} & \cos\psi_{i} \end{array}\right).$$

Thus,

(4) $$\begin{array}{cc} \sigma_{xx}^{\left(i\right)}\; & =\sigma_{x}\cos^{2}\psi_{i}+\sigma_{y}\sin^{2}\psi_{i},\\ \sigma_{yy}^{\left(i\right)}\; & =\sigma_{x}\sin^{2}\psi_{i}+\sigma_{y}\cos^{2}\psi_{i},\\ \sigma_{xy}^{\left(i\right)}=\sigma_{yx}^{\left(i\right)}\; & =\left(\sigma_{x}-\sigma_{y}\right)\sin\psi_{i}\cos\psi_{i}. \end{array}$$

To make the role of twist explicit, we consider the one-parameter symmetric orientation family

(5) $$\psi_{1}=-\frac{\pi }{2},\;\psi_{2}=+\alpha ,\;\psi_{3}=-\alpha ,$$

where α controls the relative orientation of the second and third sheets with respect to the fixed first sheet. Introducing $\bar{\sigma }=\left(\sigma_{x}+\sigma_{y}\right)/2$ and $\Delta \sigma =\sigma_{x}-\sigma_{y}$, the sum of the three rotated conductivity tensors can be written as

(6) $$\sum_{i=1}^{3}\sigma_{\psi_{i}}=3\bar{\sigma }I+\frac{\Delta \sigma }{2}\left(2\cos2\alpha -1\right)\left(\begin{array}{cc} 1 & 0\\ 0 & -1 \end{array}\right).$$

Equation (6) exposes the twist-controlled anisotropic part directly. At

(7) $$\alpha =\frac{\pi }{6}=30{}^{\circ},\;\sum_{i=1}^{3}\sigma_{\psi_{i}}=\frac{3}{2}\left(\sigma_{x}+\sigma_{y}\right)I,$$

the three-sheet conductivity triad is isotropic at the tensor-sum level even though every individual sheet remains strongly anisotropic. We denote this structurally selected tensor-compensation angle by $\alpha_{c}=30{}^{\circ}$. The three-sheet arrangement is a design choice rather than a unique minimum. Two identical anisotropic sheets with orthogonal principal axes can already cancel the traceless tensor anisotropy. Here, the fixed reference sheet, the mirror-symmetric +α/−α pair, and the two dielectric spacers provide a continuous twist parameter together with two propagation phases for full-Jones matching. Equation (7) therefore identifies a convenient tensor-compensation point rather than a uniqueness condition. Because the sheets are spatially separated, the transmitted field also depends on spacer phases and multiple reflections, so the exact matching angle $\alpha_{0}$ need not coincide with $\alpha_{c}$, although it remains close to this configuration.

### 2.2. 4 × 4 Transfer Matrix of the Twisted Three-Sheet Stack

The optical response of the multilayer structure is calculated using the standard 4 × 4 transfer-matrix method for anisotropic conducting interfaces [34,35,36]. The four homogeneous regions are indexed by j=0,1,2,3, and sheet *i* lies between regions i−1 and *i*. Regions 1 and 2 are the dielectric spacers of thicknesses $d_{1}$ and $d_{2}$, respectively.

For each plane-wave component, we use the local (p,s) basis associated with its plane of incidence and define

(8) $$a_{j}={\left(E_{p}^{+},E_{p}^{-},E_{s}^{+},E_{s}^{-}\right)}^{T},$$

where the superscripts + and − denote propagation along the +z and −z directions. The polar angle in region *j*, measured from the +z axis, is denoted by $\theta_{j}$. For an isotropic region *j* and an anisotropic conducting sheet *i*,

(9) $$D_{j}=\left(\begin{array}{cccc} c_{j} & c_{j} & 0 & 0\\ 0 & 0 & 1 & 1\\ 0 & 0 & -c_{j}/Z_{j} & c_{j}/Z_{j}\\ 1/Z_{j} & -1/Z_{j} & 0 & 0 \end{array}\right),\;W_{i}=\left(\begin{array}{cccc} 1 & 0 & 0 & 0\\ 0 & 1 & 0 & 0\\ \sigma_{sp}^{\left(i\right)} & \sigma_{ss}^{\left(i\right)} & 1 & 0\\ -\sigma_{pp}^{\left(i\right)} & -\sigma_{ps}^{\left(i\right)} & 0 & 1 \end{array}\right),$$

where $c_{j}=\cos\theta_{j}$, $Z_{j}=Z_{0}/n_{j}$, and $k_{z,j}=n_{j}k_{0}\cos\theta_{j}$. The quantities $\sigma_{pp}^{\left(i\right)}$, $\sigma_{ps}^{\left(i\right)}$, $\sigma_{sp}^{\left(i\right)}$, and $\sigma_{ss}^{\left(i\right)}$ are the conductivity components in the local (p,s) basis; their relation to the fixed laboratory-frame tensor is given in Equation (19).

The transfer matrix across sheet *i* is

(10) $$M_{i}=D_{i-1}^{-1}W_{i}^{-1}D_{i}.$$

For a dielectric spacer of thickness $d_{j}$, the propagation matrix is

(11) $$P_{j}=\mathrm{diag}\left(e^{-ik_{z,j}d_{j}},e^{+ik_{z,j}d_{j}},e^{-ik_{z,j}d_{j}},e^{+ik_{z,j}d_{j}}\right).$$

The signs in Equation (11) follow from the present transfer direction: although a physical forward wave varies as $e^{+ik_{z}z}$ for the convention $e^{-i\omega t}$, the transfer matrix maps amplitudes on the right side of a spacer back to those on the left side and therefore contains the inverse propagation factors. The total transfer matrix is

(12) $$M=M_{1}P_{1}M_{2}P_{2}M_{3}.$$

With no incoming wave from the output side,

(13) $$\left(\begin{array}{c} E_{p,i}\\ E_{p,r}\\ E_{s,i}\\ E_{s,r} \end{array}\right)=M\left(\begin{array}{c} E_{p,t}\\ 0\\ E_{s,t}\\ 0 \end{array}\right).$$

Defining

(14) $$M_{f}=\left(\begin{array}{cc} M_{11} & M_{13}\\ M_{31} & M_{33} \end{array}\right),\;M_{b}=\left(\begin{array}{cc} M_{21} & M_{23}\\ M_{41} & M_{43} \end{array}\right),$$

the transmission and reflection Jones matrices in the local (p,s) basis are

(15) $$T_{\mathrm{tot}}=M_{f}^{-1},\;R_{\mathrm{tot}}=M_{b}M_{f}^{-1},$$

with

(16) $$T_{\mathrm{tot}}=\left(\begin{array}{cc} t_{pp} & t_{ps}\\ t_{sp} & t_{ss} \end{array}\right),\;R_{\mathrm{tot}}=\left(\begin{array}{cc} r_{pp} & r_{ps}\\ r_{sp} & r_{ss} \end{array}\right).$$

### 2.3. Helicity-Resolved Angular Spectrum and Second-Order Differentiation

A finite-width beam propagating along the *z* axis is represented as a coherent superposition of plane-wave components with different transverse wave vectors [19,37]. The transmitted field is

(17) $$E_{\mathrm{out}}\left(x,y\right)=\int \int \;T\left(k_{x},k_{y}\right)\;{\tilde{E}}_{\mathrm{in}}\left(k_{x},k_{y}\right)\;e^{i(k_{x}x+k_{y}y)}\frac{dk_{x}dk_{y}}{{\left(2\pi \right)}^{2}},$$

where $T(k_{x},k_{y})$ acts on the laboratory transverse components ${\left(E_{x},E_{y}\right)}^{T}$.

For a noncentral spectral component,

(18) $$k_{r}=\sqrt{k_{x}^{2}+k_{y}^{2}},\;\varphi =\mathrm{atan}2\left(k_{y},k_{x}\right),\;k_{r}=n_{j}k_{0}\sin\theta_{j},$$

where φ is the spectral azimuth measured from the laboratory *x* axis, whereas $\psi_{i}$ remains the fixed material-axis orientation. Since the local plane of incidence rotates with φ, the conductivity tensor entering Equation (9) is

(19) $$\sigma_{ps}^{\left(i\right)}\left(\varphi \right)=U^{T}\left(\varphi \right)\sigma_{\psi_{i}}U\left(\varphi \right)=U\left(\psi_{i}-\varphi \right)\sigma_{0}U^{T}\left(\psi_{i}-\varphi \right).$$

Thus, the relevant orientation of each angular-spectrum component is the relative angle $\psi_{i}-\varphi$.

The local (p,s) amplitudes are related to the laboratory transverse components by

(20) $${\left(\begin{array}{c} E_{x}\\ E_{y} \end{array}\right)}_{\;j}=C_{j}\left(\theta_{j},\varphi \right){\left(\begin{array}{c} E_{p}\\ E_{s} \end{array}\right)}_{\;j},\;C_{j}\left(\theta_{j},\varphi \right)=U\left(\varphi \right)\left(\begin{array}{cc} \cos\theta_{j} & 0\\ 0 & 1 \end{array}\right).$$

Combining Equations (15) and (20) gives the Jones matrix in the laboratory transverse basis,

(21) $$T\left(k_{x},k_{y}\right)=C_{\mathrm{out}}\;T_{\mathrm{tot}}\;C_{\mathrm{in}}^{-1}=\left(\begin{array}{cc} t_{xx} & t_{xy}\\ t_{yx} & t_{yy} \end{array}\right).$$

We next introduce the circular-polarization basis

(22) $${\hat{e}}_{L}=\frac{{\hat{e}}_{x}+i{\hat{e}}_{y}}{\sqrt{2}},\;{\hat{e}}_{R}=\frac{{\hat{e}}_{x}-i{\hat{e}}_{y}}{\sqrt{2}},\;\left(\begin{array}{c} E_{x}\\ E_{y} \end{array}\right)=S\left(\begin{array}{c} E_{L}\\ E_{R} \end{array}\right),\;S=\frac{1}{\sqrt{2}}\left(\begin{array}{cc} 1 & 1\\ i & -i \end{array}\right).$$

In the ordered circular basis (L,R),

(23) $$\begin{array}{cc} T_{c}\; & =S^{-1}TS\\ \; & =\frac{1}{2}\left(\begin{array}{cc} t_{xx}+t_{yy}+i\left(t_{xy}-t_{yx}\right)\; & t_{xx}-t_{yy}-i\left(t_{xy}+t_{yx}\right)\\ t_{xx}-t_{yy}+i\left(t_{xy}+t_{yx}\right)\; & t_{xx}+t_{yy}-i\left(t_{xy}-t_{yx}\right) \end{array}\right)\\ \; & \equiv \left(\begin{array}{cc} A_{L\leftarrow L} & B_{L\leftarrow R}\\ B_{R\leftarrow L} & A_{R\leftarrow R} \end{array}\right). \end{array}$$

The diagonal elements $A_{L\leftarrow L}$ and $A_{R\leftarrow R}$ describe co-helicity transmission, whereas the off-diagonal elements $B_{R\leftarrow L}$ and $B_{L\leftarrow R}$ describe the two spin-flipped channels. The differentiation scheme uses circularly polarized incidence followed by postselection of the opposite circular polarization. Therefore, the scalar transfer function relevant to the differentiated output is the corresponding off-diagonal element *B*, while the co-helicity component is rejected by the output polarization analyzer.

The normal-incidence operating point is determined jointly by the symmetric twist configuration and the two spacer phases. Guided by the tensor-compensation condition of Equation (7), the spacer phases can realize a matching point at a twist angle $\alpha =\alpha_{0}$ within the symmetric family of Equation (5), with $\alpha_{0}$ lying close to $\alpha_{c}=30{}^{\circ}$. At this point, the full Jones matrix satisfies

(24) $$T\left(0,0;\alpha_{0}\right)=t_{0}I.$$

Consequently,

(25) $$A_{L\leftarrow L}\left(0,0\right)=A_{R\leftarrow R}\left(0,0\right)=t_{0},\;B_{R\leftarrow L}\left(0,0\right)=B_{L\leftarrow R}\left(0,0\right)=0.$$

Equation (25) clarifies the role of the polarization postselection: the total transmission does not vanish at the operating point. Instead, the co-helicity channel remains bright, whereas the selected spin-flipped channel has a dark spectral origin.

The quadratic order follows from the in-plane twofold symmetry. Because the optical axis of each b-As sheet is a director, $\psi_{i}$ and $\psi_{i}+\pi$ describe the same conductivity tensor. Under $\left(k_{x},k_{y}\right)\to \left(-k_{x},-k_{y}\right)$, one has φ→φ+π. Equation (19) is unchanged, while $C_{j}\left(\varphi +\pi \right)=-C_{j}\left(\varphi \right)$; the two signs cancel between the input and output transformations in Equation (21). Hence,

(26) $$T\left(k_{x},k_{y};\alpha \right)=T\left(-k_{x},-k_{y};\alpha \right),$$

so that no first-order terms in $k_{x}$ or $k_{y}$ are allowed. For a general twist parameter α, the spin-flipped channels therefore take the form

(27) $$\begin{array}{cc} B_{R\leftarrow L}\left(k_{r},\varphi ;\alpha \right)\; & =B_{R\leftarrow L}^{\left(0\right)}\left(\alpha \right)+\frac{k_{r}^{2}}{k_{0}^{2}}\left[\beta_{+2}\left(\alpha \right)e^{+2i\varphi }+\beta_{0}\left(\alpha \right)+\beta_{-2}\left(\alpha \right)e^{-2i\varphi }\right]+O\;\left(\frac{k_{r}^{4}}{k_{0}^{4}}\right),\\ B_{L\leftarrow R}\left(k_{r},\varphi ;\alpha \right)\; & =B_{L\leftarrow R}^{\left(0\right)}\left(\alpha \right)+\frac{k_{r}^{2}}{k_{0}^{2}}\left[{\tilde{\beta }}_{+2}\left(\alpha \right)e^{+2i\varphi }+{\tilde{\beta }}_{0}\left(\alpha \right)+{\tilde{\beta }}_{-2}\left(\alpha \right)e^{-2i\varphi }\right]+O\;\left(\frac{k_{r}^{4}}{k_{0}^{4}}\right). \end{array}$$

Here, $B_{R\leftarrow L}^{\left(0\right)}\left(\alpha \right)\equiv B_{R\leftarrow L}\left(0,0;\alpha \right)$ and $B_{L\leftarrow R}^{\left(0\right)}\left(\alpha \right)\equiv B_{L\leftarrow R}\left(0,0;\alpha \right)$ denote the zeroth-order spin-flipped amplitudes. The twist dependence in Equation (27) is central to the present scheme. Both the zeroth-order terms and the quadratic coefficients vary with α, but the most important effect near the operating point is the suppression of the momentum-independent background. Away from the matching configuration, the zeroth-order term produces an undifferentiated contribution in the opposite-helicity channel. As α approaches $\alpha_{0}$, this contribution is suppressed; at the matching point, Equation (25) gives $B_{R\leftarrow L}^{\left(0\right)}=B_{L\leftarrow R}^{\left(0\right)}=0$, and the quadratic term becomes the leading response. Twist therefore controls the transition from a finite zeroth-order background to a second-order-dominated spin-flipped transfer function.

At the symmetric matching point $\alpha =\alpha_{0}$, the helicity-2 terms dominate the quadratic response, giving

(28) $$\begin{array}{cc} B_{R\leftarrow L}\left(k_{x},k_{y};\alpha_{0}\right)\; & ≃\beta_{+2}\left(\alpha_{0}\right)\frac{{\left(k_{x}+ik_{y}\right)}^{2}}{k_{0}^{2}},\\ B_{L\leftarrow R}\left(k_{x},k_{y};\alpha_{0}\right)\; & ≃{\tilde{\beta }}_{-2}\left(\alpha_{0}\right)\frac{{\left(k_{x}-ik_{y}\right)}^{2}}{k_{0}^{2}}. \end{array}$$

Using the Fourier convention in Equation (17), these transfer functions correspond, apart from an overall complex factor, to

(29) $$E_{R}^{\mathrm{out}}\propto {\left(\partial_{x}+i\partial_{y}\right)}^{2}E_{L}^{\mathrm{in}},\;E_{L}^{\mathrm{out}}\propto {\left(\partial_{x}-i\partial_{y}\right)}^{2}E_{R}^{\mathrm{in}}.$$

The converted field therefore exhibits the characteristic second-order azimuthal winding with vortex charge *ℓ* = ±2.

The origin of this azimuthal phase can be understood from the rotation of the polarization basis. With the convention of Equation (22), the circular states satisfy

(30) $$U\left(\varphi \right){\hat{e}}_{\sigma }=e^{-i\sigma \varphi }{\hat{e}}_{\sigma },\;\sigma =\left\{\begin{array}{cc} +1, & L,\\ -1, & R. \end{array}\right.$$

In the paraxial limit, $C_{j}\to U\left(\varphi \right)$, so the basis-rotation contribution to a spin-flipped matrix element can be written as

(31) $$B_{-\sigma \leftarrow \sigma }\left(k_{r},\varphi \right)=e^{2i\sigma \varphi }\;A_{\sigma }\left(k_{r},\varphi \right),$$

where $A_{\sigma }$ contains the material-dependent amplitude and dynamical phase. The factor $e^{2i\sigma \varphi }$ is the Pancharatnam–Berry geometric phase associated with spin reversal [19,38]. Equivalently,

(32) $$\Delta \gamma_{\mathrm{PB}}=\arg B_{-\sigma \leftarrow \sigma }-\arg A_{\sigma }=2\sigma \varphi =-\frac{\Omega_{\sigma }}{2},$$

where $\Omega_{\sigma }$ is the oriented solid angle enclosed by the corresponding polarization path on the Poincaré sphere. The geometric-phase factor itself does not require an anisotropic sheet. For an in-plane isotropic stratified system one obtains

(33) $$B_{R\leftarrow L}^{\mathrm{iso}}=\frac{t_{p}-t_{s}}{2}e^{+2i\varphi },\;B_{L\leftarrow R}^{\mathrm{iso}}=\frac{t_{p}-t_{s}}{2}e^{-2i\varphi },$$

and rotational symmetry gives $t_{p}-t_{s}=O\left(k_{r}^{2}\right)$ near normal incidence. A bare isotropic dielectric stack can therefore already support the generic $q^{2}e^{\pm 2i\varphi }$ helicity-flipped response. In the present three-sheet configuration, the principal role of the b-As twist is instead to control the zeroth-order anisotropic Jones mismatch and to suppress $B^{\left(0\right)}$ near the operating point. The twofold symmetry removes the linear terms, while spin reversal supplies the $e^{\pm 2i\varphi }$ geometric-phase winding. The relative importance of the dielectric baseline and the b-As sheets is quantified explicitly in Section 3.2.

## 3. Results and Discussion

### 3.1. Material Response and Twist-Controlled Operating Point

**Figure 2:**
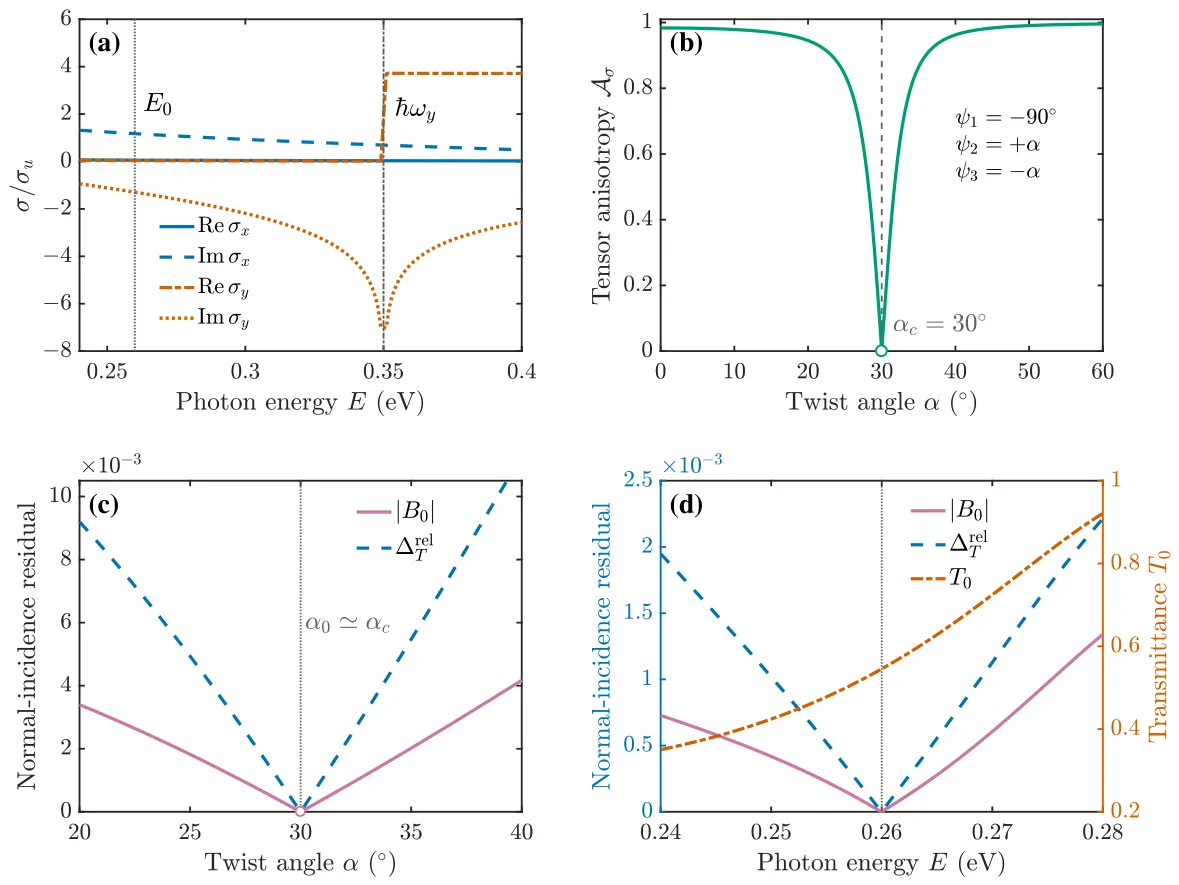
Material response and twist-controlled normal-incidence matching of the three-sheet b-As stack. (**a**) Real and imaginary parts of the principal conductivities $\sigma_{x}$ and $\sigma_{y}$, normalized by $\sigma_{u}=e^{2}/\left(4\hbar \right)$. The vertical markers indicate the operating photon energy $E_{0}$ and the *y*-direction interband onset $\hbar \omega_{y}$. (**b**) Normalized tensor anisotropy $A_{\sigma }$ of the symmetric twist family $\left(\psi_{1},\psi_{2},\psi_{3}\right)=\left(-90{}^{\circ},+\alpha ,-\alpha \right)$. The anisotropic part vanishes at the tensor-compensation angle $\alpha_{c}=30{}^{\circ}$. (**c**) Full-transfer-matrix normal-incidence response as a function of α at fixed $E_{0}$ and fixed spacer thicknesses. Both the spin-flipped background $|B_{0}|$ and the relative Jones-matrix residual $\Delta_{T}^{\mathrm{rel}}$ are strongly suppressed near $\alpha_{0}≃30.0{}^{\circ}≃\alpha_{c}$, connecting the tensor-level compensation in panel (**b**) to the optical matching condition. At $E_{0}$, $d_{2}$ satisfies $n_{s}d_{2}≃\lambda_{0}/2$. (**d**) Spectral response of the same fixed geometry. The left axis shows $|B_{0}|$ and $\Delta_{T}^{\mathrm{rel}}$, while the right axis gives the co-helicity normal-incidence transmittance $T_{0}={\left|t_{0}\right|}^{2}$. The vertical dotted line marks $E_{0}$.

We first examine the material anisotropy and the role of the symmetric twist parameter in establishing the transmission operating point. Figure 2 collects the corresponding material- and multilayer-level responses. Unless otherwise stated, the representative sheet carrier density is $n_{2D}=5.0\times 10^{17}\;m^{-2}$ ($5.0\times 10^{13}\;{\mathrm{cm}}^{-2}$). The effective masses $m_{x}=0.40m_{0}$ and $m_{y}=0.61m_{0}$ are adopted from the experimentally extracted valence-band masses of b-As along its principal in-plane directions [30]. The remaining conductivity parameters, ℏΓ=10meV, $\hbar \omega_{x}=1.00\;\mathrm{eV}$, $\hbar \omega_{y}=0.35\;\mathrm{eV}$, $s_{x}=1.7$, and $s_{y}=3.7$, are used as a representative phenomenological parameter set of the minimal anisotropic two-dimensional conductivity model [29]; they are not interpreted as independently measured optical constants of b-As. The strong in-plane optical and electrical anisotropy motivating this effective description is supported by experiments on b-As [30,31].

For the numerical calculations, the incident and output media are air ($n_{0}=n_{3}=1$), and the two spacers are modeled with $n_{s}=3.4$. We use $E_{0}=0.260\;\mathrm{eV}$ and nominal spacer thicknesses $d_{1}≃0.561\;\mu m$ and $d_{2}≃0.701\;\mu m$. At this energy, $\lambda_{0}≃4.77\;\mu m$, and the second spacer approximately satisfies the half-wave condition $n_{s}d_{2}≃\lambda_{0}/2$. For the nominal spacer configuration, the numerically optimized matching angle is $\alpha_{0}≃30.00{}^{\circ}$, with $\min|B_{0}|≃1.01\times 10^{-6}$. This remains close to the tensor-compensation angle $\alpha_{c}=30{}^{\circ}$. The half-wave condition makes the normal-incidence fields at sheets 2 and 3 nearly equivalent up to a global phase and therefore assists cancellation of their oppositely rotated off-diagonal conductivity components.

To quantify the tensor-level compensation introduced in Section 2.1, we define the normalized anisotropic part of the summed sheet conductivity as

(34) $$A_{\sigma }=\frac{{∥\sum_{i=1}^{3}\sigma_{\psi_{i}}-\frac{1}{2}\mathrm{Tr}\left(\sum_{i=1}^{3}\sigma_{\psi_{i}}\right)I∥}_{F}}{{∥\sum_{i=1}^{3}\sigma_{\psi_{i}}∥}_{F}}.$$

For the symmetric twist family $\left(\psi_{1},\psi_{2},\psi_{3}\right)=\left(-90{}^{\circ},+\alpha ,-\alpha \right)$, Equation (6) gives the tensor-compensation angle $\alpha_{c}=30{}^{\circ}$, at which $A_{\sigma }=0$.

For the complete multilayer response, we characterize the departure from the normal-incidence matching condition of Equation (24) by the relative Jones-matrix residual

(35) $$\Delta_{T}^{\mathrm{rel}}=\frac{\sqrt{|t_{xx}-t_{yy}{|}^{2}+|t_{xy}{|}^{2}+{\left|t_{yx}\right|}^{2}}}{|t_{0}|},\;t_{0}=\frac{t_{xx}+t_{yy}}{2},$$

together with the spin-flipped normal-incidence amplitude $B_{0}\equiv B_{R\leftarrow L}\left(0,0\right)$. These two quantities characterize the normal-incidence departure from the matching condition as the twist angle or photon energy is varied.

Figure 2a shows that the two principal conductivity components are strongly anisotropic in the spectral range of interest. At $E_{0}$, the imaginary parts of $\sigma_{x}$ and $\sigma_{y}$ have opposite signs, providing a pronounced in-plane anisotropic response for each individual b-As sheet. The purpose of the twist is not to eliminate the anisotropy of an individual sheet. Instead, the three rotated conductivity tensors compensate one another at the structural level. This is seen directly in Figure 2b, where $A_{\sigma }$ vanishes at $\alpha_{c}=30{}^{\circ}$, in agreement with Equation (7).

Because the sheets are separated by finite dielectric spacers, the tensor-compensation condition alone does not determine the complete optical response. The propagation phases and multiple reflections must also be included. Figure 2c therefore shows the full transfer-matrix response as the symmetric twist parameter is varied. The simultaneous suppression of $|B_{0}|$ and $\Delta_{T}^{\mathrm{rel}}$ near 30° shows that the optical matching angle $\alpha_{0}$ remains closely tied to the analytically selected tensor-compensation angle $\alpha_{c}$. The twist therefore acts primarily by suppressing the zeroth-order spin-flipped background and exposing the higher-order angular-spectrum response.

Figure 2d shows the corresponding spectral behavior with $d_{1}$ and $d_{2}$ held fixed. Around $E_{0}$, both normal-incidence residual quantities are strongly suppressed while a finite co-helicity transmission $T_{0}$ is retained. This is the situation described by Equations (24) and (25): the total transmitted field remains finite, whereas the postselected spin-flipped channel develops a dark spectral origin. The transverse-momentum dependence of this selected channel is examined next.

The half-wave interpretation also makes the fabrication sensitivity more transparent. Re-optimizing the matching angle after detuning $d_{2}$ gives the values in Table 1. A ±1% thickness change shifts $\alpha_{0}$ to approximately 30.22° and 29.77°, respectively, and raises the optimized residual $|B_{0}|$ to the $10^{-4}$ level. For ±5% detuning the optimum moves to 31.23° and 28.93°, with residuals of order $4\times 10^{-4}$–$6\times 10^{-4}$. By comparison, a ±20% variation of $n_{2D}$ changes the optimized angle only from approximately 29.98° to 30.01°, while the corresponding optimized residual remains below $3\times 10^{-6}$. The matching angle is therefore much more sensitive to spacer optical thickness than to moderate carrier-density variation.

The carrier density used here should be interpreted as an effective tunable sheet density rather than as a value already demonstrated for three buried b-As sheets. For comparison, electric-double-layer gating of the related layered semiconductor black phosphorus has reached carrier densities of order $10^{14}\;{\mathrm{cm}}^{-2}$ [39]. Realizing the assumed density independently in each buried b-As sheet would nevertheless require a dedicated gating or doping architecture. Black arsenic is also sensitive to ambient moisture [32]; a practical device would therefore require inert-environment transfer and encapsulation. Complete hBN encapsulation has provided long-term air stability in closely related black-arsenic-phosphorus mid-infrared devices [40]. Such protective layers are not included in the present proof-of-principle model and would need to be included in a device-specific transfer-matrix optimization because they introduce additional optical phases.

### 3.2. Momentum-Space Second-Order Response

**Figure 3:**
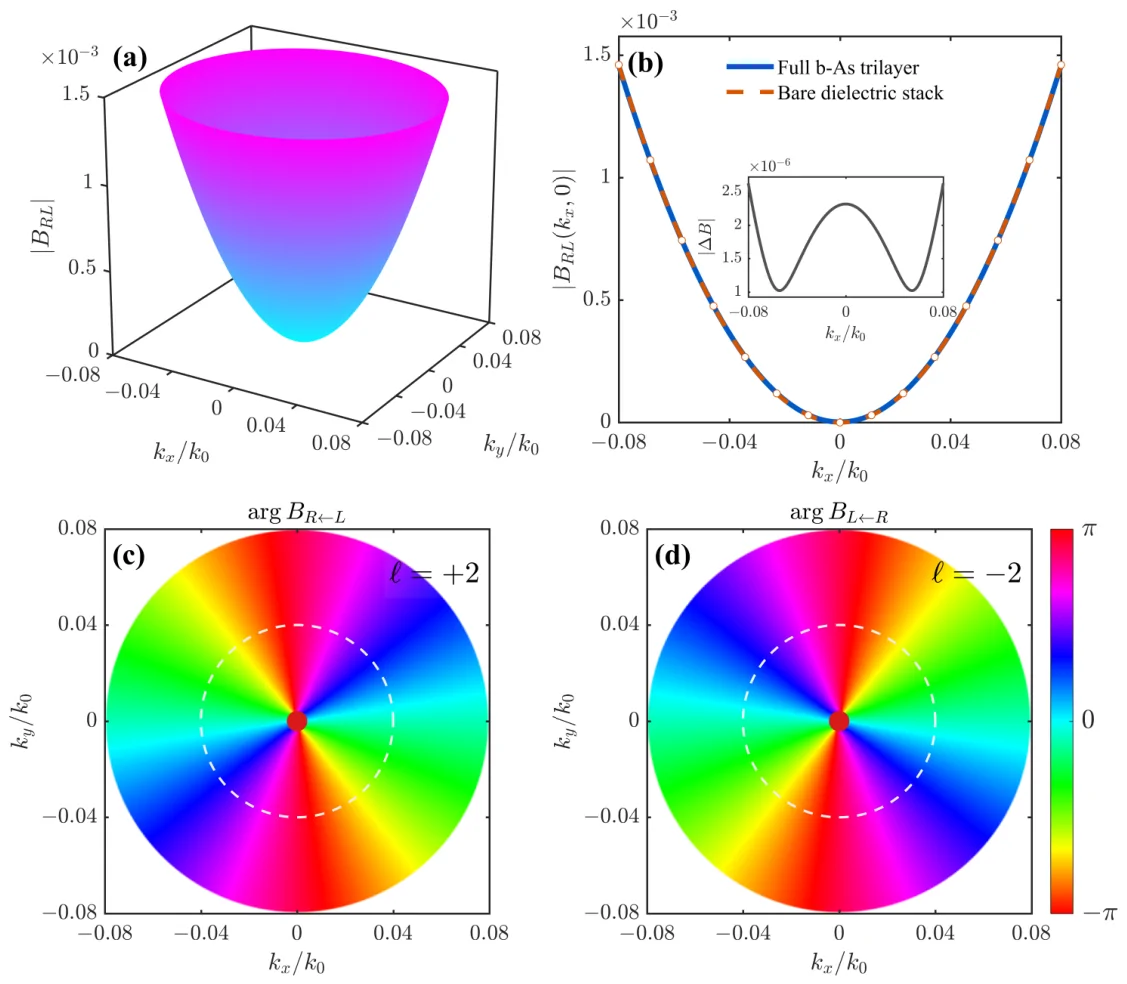
Helicity-resolved momentum-space response at $E_{0}=0.260\;\mathrm{eV}$. (**a**) Magnitude of the full spin-flipped transmission coefficient $|B_{R\leftarrow L}\left(k_{x},k_{y}\right)|$ within $k_{r}/k_{0}\leq 0.08$; the surface color follows the same magnitude and is used only to visualize its variation. (**b**) Comparison of $|B_{R\leftarrow L}\left(k_{x},0\right)|$ for the complete b-As trilayer and the corresponding bare dielectric stack with all sheet conductivities set to zero; the bare-stack result is indicated by the dashed curve with open markers. The inset shows $|B_{\mathrm{full}}-B_{\mathrm{bare}}|$. (**c**,**d**) Phase distributions of $B_{R\leftarrow L}$ and $B_{L\leftarrow R}$, showing the conjugate *ℓ* = +2 and *ℓ* = −2 windings. The white dashed circles mark a constant-$k_{r}$ contour.

Having established the twist-controlled near-matching configuration at normal incidence, we next examine the helicity-resolved transmission response in the transverse momentum space. Figure 3a shows the magnitude of the spin-flipped coefficient $B_{R\leftarrow L}\left(k_{x},k_{y}\right)$ at $E_{0}=0.260\;\mathrm{eV}$ and α=30° within the demonstrated radial aperture $k_{r}/k_{0}\leq 0.08$. A pronounced minimum appears at the spectral origin, and the response grows approximately quadratically away from it. In the small-momentum region, the leading contribution can be written as

(36) $$B_{R\leftarrow L}\left(k_{x},k_{y}\right)≃\beta_{+2}\frac{{\left(k_{x}+ik_{y}\right)}^{2}}{k_{0}^{2}},$$

with the opposite-helicity channel obeying

(37) $$B_{L\leftarrow R}\left(k_{x},k_{y}\right)≃{\tilde{\beta }}_{-2}\frac{{\left(k_{x}-ik_{y}\right)}^{2}}{k_{0}^{2}}.$$

To separate the role of the b-As sheets from the generic dielectric response, Figure 3b compares the full structure with a bare control in which all three sheet conductivities are set to zero while the two dielectric spacers are kept unchanged. At $k_{r}/k_{0}=0.08$, the two responses are nearly identical: $|B_{R\leftarrow L}{|}_{\mathrm{full}}=1.458\times 10^{-3}$ and $|B_{R\leftarrow L}{|}_{\mathrm{bare}}=1.461\times 10^{-3}$, with corresponding quadratic coefficients ${\left|\beta \right|}_{\mathrm{full}}≃0.228$ and ${\left|\beta \right|}_{\mathrm{bare}}≃0.228$. This control confirms that the dominant quadratic curvature at the selected operating point is largely inherited from the dielectric-stack p/s splitting and geometric phase described by Equation (33). The b-As sheets mainly provide the additional twist-dependent zeroth-order Jones mismatch whose suppression is shown in Figure 2c. Consistent with this interpretation, the sheet conductivities are weak on the free-space impedance scale, $Z_{0}\left|\sigma_{x}\right|≃0.027$ and $Z_{0}\left|\sigma_{y}\right|≃0.030$ at $E_{0}$. A regime in which the sheets themselves dominate the quadratic curvature would require a qualitatively stronger surface response together with re-optimization of the multilayer matching condition; such a resonance-enhanced regime lies outside the present weak-sheet design.

The angular-harmonic decomposition of Equation (27) provides a complementary measure of the quadratic response. At $q=k_{r}/k_{0}=0.01$, we obtain $|\beta_{+2}|≃0.2270$, $|\beta_{0}|≃5.6\times 10^{-4}$, and $|\beta_{-2}|≃1.6\times 10^{-6}$. The modal purity within the quadratic m=0,±2 sector,

$$P_{+2}^{\left(2\right)}=\frac{|\beta_{+2}{|}^{2}}{|\beta_{+2}{|}^{2}+|\beta_{0}{|}^{2}+{\left|\beta_{-2}\right|}^{2}},$$

is therefore approximately 99.9994% at the matching point. Away from matching, the main degradation is the re-emergence of the azimuthally invariant *ℓ* = 0 background rather than contamination by opposite winding. For example, at q=0.08 the total *ℓ* = +2 power fraction decreases to approximately 93.6%, 78.9%, and 39.1% at α=29°, 28°, and 25°, respectively. Over the sampled range 25°≤α≤35°, the combined nonzero-angular-harmonic power outside the dominant *ℓ* = +2 component remains below $10^{-8}$ of the total angular power in the numerical decomposition. Thus, the loss of total *ℓ* = +2 fraction away from matching is dominated by the restored *ℓ* = 0 background rather than by higher-winding contamination.

Figure 3c,d show the phase distributions of the two spin-flipped channels. Their opposite azimuthal windings correspond to *ℓ* = +2 and *ℓ* = −2, respectively,

$$B_{R\leftarrow L}\propto k_{r}^{2}e^{+2i\varphi },\;B_{L\leftarrow R}\propto k_{r}^{2}e^{-2i\varphi }.$$

The radial range used in Figure 3 is deliberately conservative. We quantify the validity of the second-order expansion by the angular RMS error

$$\epsilon_{2}\left(q\right)={\left[\frac{\langle |B_{\mathrm{full}}-B^{\left(2\right)}{{|}^{2}\rangle }_{\varphi }}{\langle |B_{\mathrm{full}}-B_{0}{{|}^{2}\rangle }_{\varphi }}\right]}^{1/2},$$

where $B^{\left(2\right)}$ retains the m=0,±2 quadratic coefficients extracted near the origin. At q=0.08, $\epsilon_{2}=0.48\%$; the error reaches 1%, 2%, 5%, and 10% at approximately q=0.116, 0.163, 0.259, and 0.370, respectively. We therefore use q≤0.08 as the demonstrated functional numerical aperture. With a representative criterion requiring a small residual background and at least 99% *ℓ* = +2 content at q=0.08, the useful spectral interval is approximately 0.2570–0.2625eV, corresponding to a width of 5.5meV. This interval is a performance criterion for the present design rather than an intrinsic material linewidth, and should therefore not be compared directly with bandwidths defined using different fidelity or contrast thresholds.

### 3.3. Real-Space Second-Order Differentiation and Practical Throughput

The dominant +2 harmonic identified above corresponds to the effective quadratic kernel

(38) $$H_{\mathrm{eff}}\left(k_{x},k_{y}\right)\propto {\left(k_{x}+ik_{y}\right)}^{2}=\left(k_{x}^{2}-k_{y}^{2}\right)+2ik_{x}k_{y},$$

and therefore to the real-space operator

(39) $${\hat{D}}_{+}^{\left(2\right)}={\left(\partial_{x}+i\partial_{y}\right)}^{2}=\left(\partial_{x}^{2}-\partial_{y}^{2}\right)+2i\partial_{x}\partial_{y}.$$

**Figure 4:**
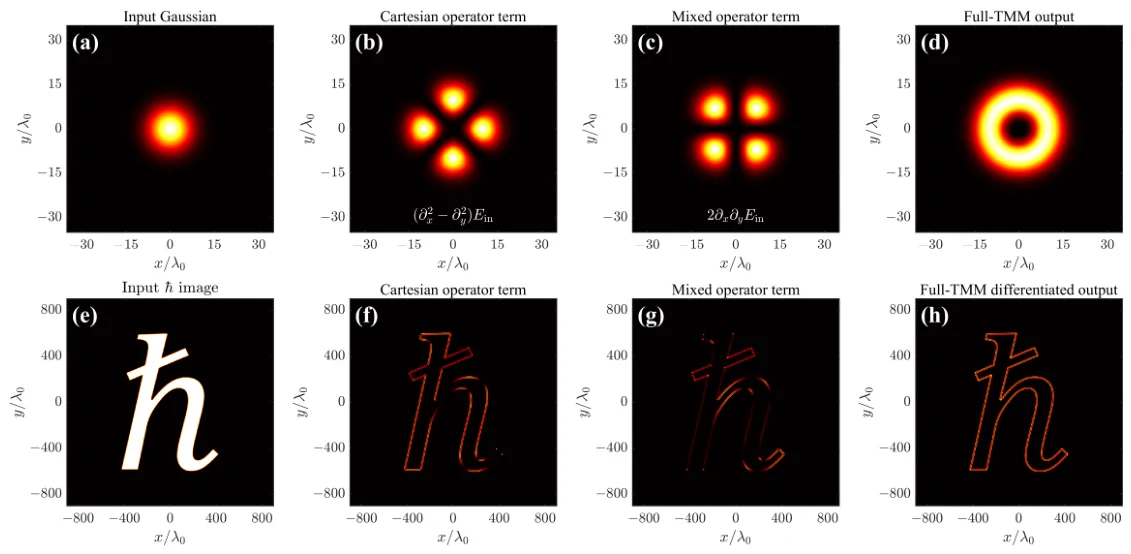
Real-space action of the second-order operator and the corresponding full-transfer-matrix response. (**a**) Input Gaussian beam with $w_{0}=10\lambda_{0}$. (**b**,**c**) Intensity patterns associated with the Cartesian $\left(\partial_{x}^{2}-\partial_{y}^{2}\right)E_{\mathrm{in}}$ and mixed $2\partial_{x}\partial_{y}E_{\mathrm{in}}$ terms of the ideal complex operator. (**d**) Physical helicity-flipped intensity obtained by propagating the same Gaussian input through the complete $B_{R\leftarrow L}\left(k_{x},k_{y}\right)$ response. (**e**–**h**) Corresponding results for the *ℏ* test image: input, Cartesian operator term, mixed operator term, and full-TMM differentiated output. Panels (**b**,**c**,**f**,**g**) are shown only as a mathematical decomposition of ${\left(\partial_{x}+i\partial_{y}\right)}^{2}$ and do not represent independent directly detected output channels. The same circular aperture $k_{r}/k_{0}\leq 0.08$ is used throughout, and the displayed intensities are independently normalized for visualization. Absolute conversion efficiencies are given in the text.

Equation (39) separates the complex second-order operator into a Cartesian term, $\partial_{x}^{2}-\partial_{y}^{2}$, and a mixed term, $2\partial_{x}\partial_{y}$, in quadrature. For a locally one-dimensional edge whose normal forms an angle θ with the *x* axis, the two terms scale as cos2θ and sin2θ, respectively. Their coherent combination therefore carries the angular factor $e^{i2\theta }$, explaining why the two decomposed terms emphasize complementary edge orientations while the full complex operator produces nearly orientation-independent second-order edge enhancement. The separated terms are used below only to visualize the operator structure and do not represent independent directly detected output channels. If separate reconstruction of these quadratures were desired experimentally, the complex helicity-flipped field could be recovered by phase-sensitive interferometry with a coherent reference, from which its real and imaginary quadratures can be reconstructed. The directly detected quantity considered here is the total helicity-flipped intensity shown in panels (d) and (h) of Figure 4.

For the device-level calculation, the output is obtained directly from the full multilayer transfer function rather than from the ideal kernel,

(40) $$E_{R}^{\mathrm{out}}\left(x,y\right)=F^{-1}\;\left[B_{R\leftarrow L}\left(k_{x},k_{y}\right)\;{\tilde{E}}_{L}^{\mathrm{in}}\left(k_{x},k_{y}\right)\right],$$

using the circular aperture $k_{r}/k_{0}\leq 0.08$. Figure 4 visualizes the Cartesian and mixed terms of the ideal operator together with the physical helicity-flipped output calculated from the complete transfer function. For a Gaussian beam with $w_{0}=10\lambda_{0}$, more than 99.999% of the input angular-spectrum power lies inside the demonstrated aperture. The normalized complex-field overlap between the full-TMM output and the complete ideal quadratic output exceeds 0.998 for both the Gaussian and *ℏ* test-image inputs, confirming that the multilayer reproduces the expected second-order spatial pattern within the stated numerical aperture.

High normalized operator fidelity does not imply high optical throughput. For a given input angular spectrum we define the postselected spin-flip efficiency as

(41) $$\eta_{\mathrm{flip}}=\frac{\;\int \int \;|B_{R\leftarrow L}\left(k_{x},k_{y}\right){\tilde{E}}_{\mathrm{in}}\left(k_{x},k_{y}\right){|}^{2}\;dk_{x}dk_{y}}{\;\int \int \;|{\tilde{E}}_{\mathrm{in}}\left(k_{x},k_{y}\right){|}^{2}\;dk_{x}dk_{y}}.$$

The Gaussian and *ℏ* inputs give $\eta_{\mathrm{flip}}=2.65\times 10^{-8}$ and $2.66\times 10^{-9}$, respectively, while the co-helicity transmission is approximately 0.55. The present operating point therefore realizes the normalized second-order operator with low absolute conversion efficiency. A nonideal circular analyzer adds leakage from the much stronger co-helicity channel. Modeling the detected field as

$$H_{\det}=B_{R\leftarrow L}+\sqrt{\mathrm{ER}}\;e^{i\delta }A_{L\leftarrow L},$$

where ER is the analyzer intensity extinction ratio, shows that an extinction ratio of $10^{-4}$ would overwhelm the desired postselected signal. Finite polarization extinction is therefore an important experimental constraint for the present weak-conversion design.

This limitation also clarifies the comparison with the anisotropic ENZ second-order differentiator of Ref. [19]. That platform was designed to enhance spin-orbit conversion resonantly and can reach much larger local conversion efficiencies, whereas the present b-As structure emphasizes twist control of the normal-incidence Jones background rather than throughput. From an optical-processing viewpoint, the useful feature of the present geometry is that the undifferentiated background can be controlled through crystallographic orientation without subwavelength patterning of the conducting sheets. The tradeoff is the additional requirement of multilayer alignment, spacer control, and polarization postselection. The main qualitative differences among these related platforms are summarized in Table 2.

## 4. Conclusions

We have investigated transmission-mode second-order spatial differentiation in a symmetric three-sheet b-As stack. Relative twist controls the normal-incidence Jones mismatch and suppresses the zeroth-order helicity-converted background, allowing a quadratic spin-flipped response with conjugate *ℓ* = ±2 phase winding to dominate near the matching condition. The corresponding complex operator takes the form ${\left(\partial_{x}\pm i\partial_{y}\right)}^{2}$, combining Cartesian and mixed second-order derivatives, and full transfer-matrix propagation confirms the expected beam shaping and two-dimensional edge enhancement.

Control calculations further show that, at the selected operating point, the dominant quadratic curvature is largely inherited from the dielectric multilayer, while the b-As sheets mainly provide the twist-controlled matching of the postselected channel. The present implementation therefore emphasizes operator control rather than conversion efficiency. Improving the sheet response and multilayer resonance, together with practical control of spacer thickness, encapsulation, and polarization extinction, offers a route toward higher-throughput implementations while retaining the twist-controlled matching functionality.

## Figures and Tables

**Table 1:** Sensitivity of the optimized normal-incidence matching condition to changes in the second spacer thickness.

Δ*d*_2_/*d*_2_	*α*_0_ (deg)	min|*B*_0_|
−5%	31.23	$3.88\times 10^{-4}$
−3%	30.71	$2.53\times 10^{-4}$
−1%	30.22	$9.15\times 10^{-5}$
0	30.00	$1.01\times 10^{-6}$
+1%	29.77	$1.02\times 10^{-4}$
+3%	29.34	$3.32\times 10^{-4}$
+5%	28.93	$6.04\times 10^{-4}$

**Table 2:** Qualitative comparison with closely related differentiation and PSHE platforms. Quantitative values are listed only when their definitions are sufficiently comparable.

Platform	Main Control Mechanism	Representative Feature	Main Tradeoff
Present three-sheet b-As stack	Symmetric twist and spacer phases control the Jones matching	High-purity second-order response within NA =0.08; no subwavelength patterning	Low postselected efficiency; multilayer alignment, spacer control, and strong polarization rejection required
Anisotropic ENZ slab [19]	Resonantly enhanced spin-orbit conversion	Much stronger local conversion; selected components can approach unity in the idealized anisotropic design	Requires an anisotropic ENZ medium and resonant operating conditions
Single-interface b-As PSHE [33]	Crystal anisotropy at one interface	Structurally simpler polarization-sensitive PSHE platform	Does not provide multilayer twist-controlled Jones matching
Twisted few-layer anisotropic 2D PSHE [34]	Relative twist and Fabry–Pérot phase	Demonstrates twist control of spin-Hall response	Not formulated as the present $B_{0}$-suppressed second-order differentiator

## Data Availability

The data presented in this study are available within the article. Additional numerical data supporting the findings of this study are available from the corresponding author upon reasonable request.
